# Incorporating Neighborhood Choice in a Model of Neighborhood Effects on Income

**DOI:** 10.1007/s13524-018-0672-9

**Published:** 2018-05-09

**Authors:** Maarten van Ham, Sanne Boschman, Matt Vogel

**Affiliations:** 1 OTB–Research for the Built Environment, Faculty of Architecture and the Built Environment, Delft University of Technology, PO Box 5043, 2600 GA Delft, The Netherlands; 2 School of Geography and Sustainable Development, University of St Andrews, Irvine Building, University of St Andrews, North Street, St Andrews, KY16 9AL Fife, Scotland UK; 3 Social and Behavioural Sciences, Utrecht University, Padualaan 14, 3584CH, Utrecht, The Netherlands; 4 Department of Criminology and Criminal Justice, University of Missouri–St. Louis, St. Louis, MO USA

**Keywords:** Neighborhood effects, Neighborhood choice, Income, Selection bias, Conditional logit

## Abstract

Studies of neighborhood effects often attempt to identify causal effects of neighborhood characteristics on individual outcomes, such as income, education, employment, and health. However, selection looms large in this line of research, and it has been argued that estimates of neighborhood effects are biased because people nonrandomly select into neighborhoods based on their preferences, income, and the availability of alternative housing. We propose a two-step framework to disentangle selection processes in the relationship between neighborhood deprivation and earnings. We model neighborhood selection using a conditional logit model, from which we derive correction terms. Driven by the recognition that most households prefer certain types of neighborhoods rather than specific areas, we employ a principle components analysis to reduce these terms into eight correction components. We use these to adjust parameter estimates from a model of subsequent neighborhood effects on individual income for the unequal probability that a household chooses to live in a particular type of neighborhood. We apply this technique to administrative data from the Netherlands. After we adjust for the differential sorting of households into certain types of neighborhoods, the effect of neighborhood income on individual income diminishes but remains significant. These results further emphasize that researchers need to be attuned to the role of selection bias when assessing the role of neighborhood effects on individual outcomes. Perhaps more importantly, the persistent effect of neighborhood deprivation on subsequent earnings suggests that neighborhood effects reflect more than the shared characteristics of neighborhood residents: place of residence partially determines economic well-being.

## Introduction

The neighborhood effects literature concerns itself with identifying causal effects of living in (deprived) neighborhoods on a range of individual-level outcomes, such as income, education, employment, and health. The literature on neighborhood effects is far from conclusive, and a major debate persists as to the size and significance of neighborhood effects as well as whether the effects are causal. Several studies have suggested that selection—not causality—is behind most of the current neighborhood-effects evidence (e.g., Bolster et al. 2007; Oreopoulos 2003; van Ham and Manley 2010; van Ham et al. 2012a). According to this perspective, many existing studies have failed to convincingly show real causal neighborhood effects because they either ignored or failed to adequately address neighborhood selection (Durlauf 2004; van Ham and Manley 2010). Thus, despite the impression that neighborhood effects are important, these studies in reality might simply show correlations between individual and neighborhood characteristics (Cheshire 2007). From this vantage point, studies claiming to have found that poor neighborhoods make people poor(er) likely show only that poor people live in poor neighborhoods because they cannot afford to live elsewhere (Cheshire 2007).

The problem with estimating neighborhood effects on, for example, individual income is that people are nonrandomly allocated to neighborhoods; people select into neighborhoods based on their preferences and resources, in combination with housing availability. That is, people tend to move to neighborhoods that have affordable dwellings, match their tenure preferences, and are associated a low likelihood of discrimination by landlords against them. As a result of this selection process, parameter estimates of neighborhood effects are likely inflated because the characteristics that drive households into certain areas are highly correlated with the outcomes of interest to most researchers. Several econometric techniques have been proposed to correct for selection effects—for example, instrumental variables or fixed-effects models that hold constant time-invariant factors that presumably vary across households. Although these techniques can reduce selection bias, no perfect fix exists with which to completely rule out threats posed by endogeneity (Boschman 2015; Harding 2003; Vogel et al. 2017). Perhaps more importantly, controlling for neighborhood selection using such approaches is suboptimal because the processes that funnel certain households into particular neighborhoods are theoretically meaningful and should be modeled explicitly (Hedman and van Ham 2012). Instead of treating neighborhood selection as a nuisance that needs to be controlled away, we present an empirical framework that directly incorporates neighborhood selection in models of neighborhood effects (see also van Ham and Manley 2012).

Few studies have attempted to model neighborhood choice to correct for selection bias in models of neighborhood effects (see Hedman and Galster 2011; Ioannides and Zabel 2008; Sari 2012). Following Ioannides and Zabel (2008), we model neighborhood choice using a conditional logit model and subsequently incorporate correction components into a neighborhood-effects model of individual income from work. This approach allows us to adjust our neighborhood-effects model for selection processes driven by various household and neighborhood characteristics that are assessed simultaneously and in combination.

Our approach diverges in two crucial ways from prior work using a similar two-step framework (Ioannides and Zabel 2008). First, we proceed by estimating a conditional logit model on the full choice set of available neighborhoods in a regional housing market versus a smaller random choice set. As we argue later, only the full choice set allows us to properly correct for nonrandom selection in the neighborhood-effects model. We next derive a series of linear probabilities from the conditional logit model that reflect the likelihood that a household will choose to move to a specific neighborhood over all alternative neighborhoods in the region. These probabilities form the basis for the correction terms used in the subsequent neighborhood-effects model. Given the high degree of collinearity among items, we employ principal components analysis (PCA) to reduce the number of correction terms in the subsequent neighborhood-effects model. The specification of the conditional logit model from which these terms are derived allows for these reduced components to be interpreted as the probability that a certain type of *household* will select into a certain type of *neighborhood*. We incorporate these components into the second-stage neighborhood-effects model to account for the differential sorting of households into particular *types of neighborhoods,* the characteristics of which are likely conflated with subsequent earnings. This approach is conceptually appealing given that most households select on neighborhood type rather than a specific neighborhood, and preferences usually vary by households’ sociodemographic characteristics. We estimate our models on longitudinal population data from the Netherlands Social Statistical Database (SSD), a population registry comprising geocoded individual-level data covering the entire population of the Netherlands from 1999 to 2013.

## Background

The body of literature on the so-called neighborhood effects—defined here as the independent influence of the residential environment on individual outcomes—has grown considerably over the last two decades (see Durlauf 2004; Ellen and Turner 1997; Galster 2002; Nieuwenhuis 2016; Nieuwenhuis and Hooimeijer 2016; Sampson et al. 2002; Sharkey and Faber 2014; van Ham and Manley 2012; van Ham et al. 2012a, b). Many studies have reported neighborhood effects on outcomes such as school dropout, childhood achievement, transition rates from welfare to work, deviant behavior, social exclusion, social mobility, and income.

Since the seminal work by Wilson (1987), theoretical explanations of neighborhood effects have been expanded to include role model effects and peer group influences, social and physical disconnection from job-finding networks, a culture of poverty leading to dysfunctional values, discrimination by employers and other gatekeepers, access to public services, and exposure to criminal behavior. For an excellent overview of potential causal mechanisms, see Galster 2012. The neighborhood-effects literature suggests that living in a low-income neighborhood, or a neighborhood with a high concentration of poverty, can have a negative effect on the incomes of individuals. Various causal mechanisms could lead to such negative contextual effects on individual incomes (Galster 2012). For example, those living in high-poverty neighborhoods could have difficulties accessing good employment opportunities due to the spatial distribution of jobs and the lack of transportation. Also, people living in high-poverty neighborhoods might lack job-finding networks that could help them to find (better) paid positions. In addition, the lack of positive role models in the residential neighborhood might lead to negative attitudes toward paid employment. People living in high-poverty neighborhoods can also face discrimination from employers, thus reducing the probability of finding a job or increasing earnings.

The concept of neighborhood effects is academically intriguing, and policymakers have embraced the concept to justify area-based policies (van Ham and Manley 2012). Despite the popularity of the concept and the ever-growing body of literature, however, considerable debate remains as to the importance of neighborhood effects above and beyond the shared characteristics of neighborhood residents. Further, although increasing evidence suggests that neighborhoods are relevant for the social and economic well-being of their residents, many studies have struggled with the identification of causal neighborhood effects because they have either ignored or failed to adequately address the forces that differentially funnel certain people into particular areas (Durlauf 2004; van Ham and Manley 2010). The main problem is that people do not choose where they live at random. The neighborhood sorting process is highly structured, and often the outcome of interest (e.g., income) may also be responsible for people selecting into deprived neighborhoods in the first place (van Ham and Manley 2012). In other words, impoverished neighborhoods may not make residents poor(er). Rather, low-income households tend to live in particular types of places—for instance, where rent is low, landlords are less discriminating, and (most importantly) housing is available (Desmond 2016). Disentangling the shared characteristics of neighborhood residents from true neighborhood effects is paramount for understanding whether and how characteristics of residential places influence individual outcomes, such as economic well-being.

A growing body of literature underscores the importance of neighborhood choice in determining the spatial distribution of households across metropolitan areas. Most households have preferences about the type of neighborhoods in which they want to live and thus concentrate their search efforts in these areas. The availability of a dwelling with the right characteristics in the housing vacancy chain then determines the exact neighborhood in which a household locates (see White 1971). Most studies have modeled the probability that a household moves to a certain type of neighborhood based on one or two neighborhood characteristics—typically, the level of deprivation and/or the level of concentration of ethnic minorities (Bråmå 2006; Clark and Ledwith 2007; Logan and Alba 1993). Capturing neighborhood selection with only one or two characteristics does little justice to the variety of neighborhoods in the urban housing market. Hedman et al. (2011; see also Boschman and van Ham 2015; Sermons 2000) took a different approach. Following Ioannides and Zabel (2008) and Quillian and Bruch (2010), they applied a conditional logit model (McFadden 1974) that allowed for multiple characteristics of destination neighborhoods to be assessed simultaneously and in combination. The conditional logit model estimates the probability that a household chooses a certain neighborhood from a set of alternative neighborhoods, based on interaction effects between household characteristics and a range of neighborhood characteristics. Using administrative data from Sweden, Hedman et al. (2011) reported that neighborhood sorting is a highly structured process. Households were more likely to choose neighborhoods where the population composition matched their own social and demographic backgrounds. Income was the most important driver of the sorting process: higher-income households were most likely to sort into high-income neighborhoods, and low-income households were most likely to sort into low-income neighborhoods. However, other socioeconomic and demographic characteristics were also important. Ethnic minorities moved to neighborhoods with higher shares of ethnic minorities, and families with children to neighborhoods with many families with children. As a result of the neighborhood choices made by moving households, neighborhood characteristics were reproduced over time. Hedman et al. (2011:1395) were careful to note that

[T]he concept of choice needs to be used with caution. Households make choices within a restricted choice set. Choices are restricted by household preferences, resources, and restrictions, but also by constraints imposed by the structure of the housing market. It is very likely that poor households do not “choose” to move to poverty neighbourhoods, but move there because they cannot afford to live anywhere else.

Consistent with this observation, van Ham and Manley (2012) argued that one of the most pressing challenges for research on neighborhood effects is to explicitly incorporate neighborhood selection in models of neighborhood effects. Controlling for selection effects through econometric modeling alone may not be sufficient because selection is at the very heart of understanding neighborhood effects. They further advocated for the necessity of a theory of selection bias to help explicate the “unmeasured characteristics which cause people to move to certain neighborhoods, and also cause people to have a certain income, health or other outcome” (van Ham and Manley 2012:2791). Only a few studies have tried to explicitly model neighborhood choice itself and to use the outcomes to correct for bias in models of neighborhood effects (see, e.g., Hedman and Galster 2011; Ioannides and Zabel 2008; Sari 2012). Although several studies have attempted to address the sorting problem, we will briefly discuss three different approaches.

Hedman and Galster (2011) specified a structural equation model in which both neighborhood income mix (neighborhood sorting) and individual income (neighborhood effects) were modeled as mutually reinforcing. This approach was designed to avoid both selection on unobservable characteristics and endogeneity resulting from nonrandom neighborhood selection. Their results suggested that models failing to control for endogeneity underestimate the true neighborhood effect. In other words, the parameter estimates for neighborhood effects were smaller in the models that did not correct for selection bias. This seems somewhat counterintuitive: one would expect that controlling for selection would reduce the effect of neighborhood characteristics on individual outcomes.

Sari (2012) used a different approach to address the endogeneity problem that results from the fact that residential location may be jointly determined with employment status as a result of nonrandom sorting. Two models were estimated. First, a bivariate probit estimated the probability of living in a deprived neighborhood and the probability of being employed. Second, a probit model was estimated on a subsample of households living in public housing, assuming that the location choice was exogenous in this sample. The results of this approach showed that individual unemployment depends not only on experience and skills but is also related to residential location (Sari 2012).

Finally, Ioannides and Zabel (2008) developed a two-step model of housing structure demand that controlled for the nonrandom sorting into neighborhoods. The first step used a conditional logit model to model choice for a specific neighborhood from a set of alternative neighborhoods. The choice set was determined by the chosen neighborhood in which the household lived plus a sample of 10 alternative census tracts, randomly selected from all census tracts of the metropolitan area. This resulted in a choice set of 11 tracts, 10 of which were random. The conditional logit model included interactions between individual characteristics and tract-level characteristics and, similar to Hedman et al. (2011), confirmed that individuals select into tracts with neighbors like themselves. Ioannides and Zabel (2008) subsequently modeled housing structure demand and included 11 bias correction terms, one for probability of choosing each of the alternative neighborhoods in the choice set. Like findings of Hedman and Galster (2011), the results from this two-stage model demonstrated that neighborhood effects were strengthened when neighborhood choice was controlled for (Ioannides and Zabel 2008).

## Adjusting for Selection by Neighborhood Type

The current study builds on and moves beyond prior research incorporating neighborhood selection in models of neighborhood effects. Following Ioannides and Zabel (2008), we employ a two-step approach: we first model neighborhood selection and then model neighborhood effects on individual income. The linear probabilities from the first-stage model are used to adjust for the nonrandom selection of households into neighborhoods in the neighborhood-effects model.

We depart from the approach presented by Ioannides and Zabel (2008) in two important ways. First, whereas Ioannides and Zabel (2008) constructed correction terms based on the probability that a household selects a certain neighborhood, we construct correction components based on the probability that a household selects a certain *type of neighborhood*. There are both conceptual and methodological reasons to use neighborhood types to construct correction components. Conceptually, most households are likely to search for a dwelling in a particular type of neighborhood instead of a dwelling in a specific neighborhood. This is especially the case when a regional housing market is divided into a large number of smaller neighborhoods. Many of these neighborhoods will share similar characteristics, generating only a limited number of neighborhoods types. This leads us to a methodological reason to use neighborhood types to construct correction components for nonrandom selection of neighborhoods. Correction terms based on individual neighborhoods are likely to be highly intercorrelated because many neighborhoods have very similar characteristics and hence a very similar probability that a household will move there. In the Data and Methods section, we explain in greater detail how we employ a PCA to construct correction components based on neighborhood types to overcome this problem.

Second, our approach departs from Ioannides and Zabel (2008) in that our neighborhood-choice model (the first step) uses the full closed-choice set of all alternative neighborhood options within a regional housing market rather than a random subset of alternative neighborhoods. Of course, households do not actively consider all possible neighborhoods when choosing where to live: most households focus on a limited number of parameters (e.g., proximity to schools, building age, lot size) as they begin their housing search based on their (lack of) knowledge of the regional housing market. But because we do not know the types of neighborhoods where households search, we cannot make assumptions with regard to a more limited choice set. On the other hand, the Dutch housing market is extremely transparent because households have online access to information on almost all dwellings that are for sale and for rent. The majority of real estate agents advertise dwellings for sale on the website www.funda.nl, which allows households to search for both rented and owner-occupied dwellings on a map and see detailed neighborhood characteristics. The majority of socially rented dwellings are offered on the woningnet.nl website, which operates on the regional housing market level.

## Data and Methods

### Data and Research Population

Our empirical analyses draw on longitudinal population data from the Netherlands SSD, a population registry comprising geocoded individual-level data covering the entire population of the Netherlands from 1999 to 2013. We append these data to neighborhood-level information, including ethnic, household, dwelling, and income composition, compiled by Netherlands Statistics (Kerncijfers Wijken en Buurten). We focus on heads of household who moved within the Utrecht urban region during 2009. We first estimate a selection model in which household heads select their subsequent destination neighborhood based on neighborhood characteristics prior to the move (measured in 2008). We then model the effects of neighborhood characteristics after the move (measured on January 1, 2010) on subsequent income from work in 2013.

Our decision to focus on the Utrecht urban region is twofold. First, the neighborhood selection model necessitates a study area that functions as a single regional housing market to ensure that (at least in theory) all neighborhoods within this area are part of the choice set of moving households. Second, we want an area with a large variation in neighborhood types. The Utrecht urban region, which consists of the city of Utrecht and the surrounding suburban municipalities, meets these criteria. In the Netherlands, more than 70 % of moves are within urban regions (Vliegen 2005). Within the Utrecht urban region, the social housing sector employs a choice-based letting system that allows applicants to bid on dwellings throughout the region (via the website www.woningnet.nl). The region is characterized by large variation in terms of ethnic composition, dwelling prices, housing tenure, and accessibility of facilities between neighborhoods. Consistent with prior research in the Netherlands, we use administrative neighborhoods (*buurten*) to reflect residential neighborhood boundaries. These neighborhoods are relatively small-scale, administratively determined geographic areas. In urban areas, these neighborhoods are analogous to the more familiar census tract in U.S.-based research, often consisting of relatively homogenous populations and covering, on average, one-half square kilometers in land area. Our initial sample contains 256 neighborhoods in Utrecht.

Based on the administrative data, we identify 25,643 household heads who lived in the Utrecht urban region on the first of January 2010 and who moved there from within the urban region after the first of January 2009, thus meeting our selection criteria. Households who moved to the Utrecht urban region from elsewhere are excluded from the analytic sample because we cannot assume that they included only neighborhoods within the Utrecht urban region in their choice set. Of the 256 neighborhoods in Utrecht, we exclude 53 because of missing data on neighborhood average income and average dwelling values. Income data are provided for only those neighborhoods with at least 200 inhabitants, and average dwelling values are provided for only those neighborhoods with at least five dwellings. Excluding these 53 neighborhoods results in the exclusion of 848 heads of household who moved to these neighborhoods. Because our modeling strategy necessitates information on the income of the household, we therefore exclude another 601 household heads for which no data are available on income. We thus have an analytic sample of 24,014 individuals who lived in 203 neighborhoods.

### Modeling Strategy

Our modeling strategy unfolds in two steps. We first estimate a conditional logit model in which all 24,014 household heads select one neighborhood from a choice set of 203 neighborhoods within the Utrecht urban region (the selection model). The model is based on interactions between personal characteristics and the characteristics of the neighborhoods in the choice set. The conditional logit model has clear advantages over alternative strategies because it allows us to address selection effects associated with multiple individual characteristics and neighborhood characteristics simultaneously. From here, we derive the linear probabilities reflecting the likelihood that a household moves to Neighborhood 1, Neighborhood 2 , . . . , Neighborhood 203. The conditional logit model on *all* potential neighborhood options allows us to retain a high degree of precision in the estimation of the conditional probabilities. Following Ioannides and Zabel (2008), we transform these linear probabilities to generate correction terms akin to the inverse Mills ratios popularized by Heckman’s (1979) two-stage regression framework. Because the conditional logit model incorporates interactions between household and neighborhood characteristics, these terms can be loosely interpreted as the probability that a certain type of household selects a particular neighborhood. Note that the neighborhood selection process is highly structured such that, for instance, young families demonstrate strong preferences for living with other young families, and ethnic minorities prefer neighborhoods with a large concentration of families with similar backgrounds. As a result, these terms tend to “hang together” for certain types of households, displaying high-levels of collinearity. We therefore employ PCA to reduce these 203 terms to a more narrow set of correction components. Given the specification of the conditional logit model from which these terms are derived, we can interpret the reduced components as reflecting the probability that a certain *type of household* will select into a certain *type of neighborhood*: for instance, minority heads of household will demonstrate a preference for living in neighborhoods with those from a similar ethnic background.

In the second step, we estimate a neighborhood-effects model in which we predict individual income from work in 2013 as a function of the characteristics of the residential neighborhood on January 1, 2010. In other words, we examine the effect of neighborhood characteristics on subsequent earnings among heads of household who moved within the Utrecht region in 2009. Our neighborhood-effects model includes the neighborhood type correction components derived from the neighborhood-selection model and the PCA. We restrict this model to heads of household who were employed in 2013 (thus excluding students, entrepreneurs, or people on welfare benefits) because the causal mechanisms that produce neighborhood effects on income will be different for employees than for other groups. Of the 24,014 household heads in the selection model, 13,430 were employed in 2013 and are therefore included in the neighborhood-effects model.

### The Selection Model

We use a conditional logit model to model neighborhood selection. In this model, household *i* selects neighborhood *j* with the highest utility from a choice set of *J* neighborhoods. The utility of a neighborhood depends on the neighborhood’s characteristics and the value of these characteristics to households, and is therefore calculated as neighborhood characteristics times parameters plus an error term (Hoffman and Duncan 1988; McFadden 1974). If we assume that the error term is identically and independently extreme value distributed across neighborhoods, the probability that household *i* chooses neighborhood *j—*thus, that the utility of neighborhood *j* to household *i* is higher than the utility of all other neighborhoods—can be estimated. Thus, let *P*_*ij*_ denote the probability that household *i* will choose neighborhood *j*, based on the characteristics of the of the *j*th neighborhood (*N*_*j*_) and the characteristics of the other neighborhoods in the choice set (*N*_*k*_). Following Hoffman and Duncan (1988), the conditional logit model is written as follows:

1 $$ {P}_{ij}=\frac{\exp \left(\upbeta {N}_j\right)}{\sum_{k=1}^J\exp \left(\upbeta {N}_k\right)}. $$

The utility of a neighborhood to a specific household depends on the match between individual and neighborhood characteristics and, thus, on the value of the neighborhood’s characteristics to the specific household. The selection of a neighborhood is modeled *within* a household; therefore, the household characteristics do not vary between neighborhood options. To include household characteristics in the model, they must be interacted with neighborhood characteristics. This can be included in Eq. (1) by letting **X**_*i*_ denote the characteristics of the *i*th household.

2 $$ {P}_{ij}=\frac{\exp \left(\upbeta {N}_j{\mathbf{X}}_i\right)}{\sum_{k=1}^J\exp \left(\upbeta {N}_k{\mathbf{X}}_i\right)}. $$

All households in our model moved during the 2009 calendar year and thus selected a new neighborhood; the selected neighborhood is the neighborhood where the household lived on January 1, 2010. When possible, we use neighborhood characteristics from 2008 in the selection models because presumably households select their neighborhood based on the characteristics of the neighborhood before they move. We model neighborhood selection based on the following neighborhood characteristics: household composition, housing characteristics (tenure composition, share of dwellings built after 2000), accessibility, dwelling values, and the share of non-Western minorities (see upcoming Table 1). Measuring neighborhood characteristics before the move is important in order to avoid endogeneity problems (Manski 1993)—that is, conflating the characteristics of in-migrants with the later composition of the neighborhood. The data on neighborhood housing characteristics are, however, available only in 2009. Therefore, we use this information as a proxy for the housing characteristics in 2008, before the move. Characteristics of moving households might affect the neighborhood ethnic and household composition, but they cannot affect the building period or tenure composition of the neighborhood.

**Table 1:** Descriptive statistics: Neighborhood characteristics (selection model), *N* = 203

	Mean	SD	Min.	Max.
Average Dwelling Values (× 1,000) (2008)	291.3	138.5	138.0	1,098.0
Restaurants Within 3 km (2008)	76.2	92.3	0	268.3
Distance to Train Station (2008)	3.9	3.4	0.3	12.2
Distance to Highway Access Lane (2008)	1.9	0.9	0.1	6.4
Share of Dwellings Built >2,000 (2009)	14.0	26.2	0	100
Share of Social Housing (2009)	30.5	24.2	0	100
Share of Private Rental (2009)	14.0	11.8	0	92
Share of Singles (2008)	41.3	18.5	10.0	97
Share of Couples (2008)	26.8	6.7	3.0	46
Share of Non-Western Minorities (2008)	12.5	12.2	0	79

We interact these neighborhood characteristics with personal characteristics to estimate differences between households in neighborhood selection. We use household characteristics of the new household, after the move (measured on January 1, 2010). If households change during a move—for instance, when two people start living together, or when an individual leaves the parental home—the characteristics of the new household (rather than the old household) determine residential preferences and therefore neighborhood selection. We assume that households do not experience any unexpected changes between the move (at some point in 2009) and January 1, 2010. A couple that selected a new neighborhood based on their shared residential preferences and opportunities, however, may be separated on January 1, 2010.

### Constructing Correction Components for Neighborhood Types

We use the conditional logit model to estimate the conditional probability that a household selects a specific neighborhood over all other alternative neighborhoods. Departing from prior research (e.g., Ioannides and Zabel 2008), we use the full choice set of available neighborhoods instead of a random sample of neighborhoods. In the appendix, we detail our method and justify the necessity of the full choice set to construct meaningful correction terms. As a robustness check, we also compare our results of the neighborhood-effects model using correction terms based on the full choice set with a model including correction terms based on a random choice set. Because households can select a neighborhood from a choice set of 203 neighborhoods, the selection model yields 203 linear probabilities per individual. These probabilities reflect the likelihood that a household head will decide to live in a given neighborhood based on his or her own sociodemographic background and the characteristics of the neighborhood in question. Similar to Ioannides and Zabel (2008), we use these predicted probabilities to generate correction terms analogous to the more familiar inverse Mills ratios popularized by Heckman’s (1979) two-stage regression framework.

When entered in the second-stage model, the 203 correction terms gleaned from the selection model are highly intercorrelated, which makes sense because the correction terms reflect the probability that certain types of people will select certain types of neighborhoods. For instance, ethnic minorities demonstrate a preference to live with other ethnic minorities, and young families prefer to live among other young families. Some households may strongly prefer a handful of neighborhoods and demonstrate an aversion to living in other types of areas. These preferences are allocated along sociodemographic lines. Thus, when all 203 correction terms are included in the second-stage model, they display high levels of collinearity because many neighborhoods share similar characteristics. This prohibits the estimation of the neighborhood-effects regression models with all correction terms entered simultaneously.

To overcome the collinearity issues, we perform a PCA to reduce the number of variables necessary to capture all variance in the correction terms (and remedy the high degree of correlation). The model produces eight principal components with eigenvalues greater than 1.0 that collectively capture 98.7 % of the total variance. These components are then orthogonally rotated to generate eight correction terms to be included in the second-stage neighborhood-effects model. As noted, the specification of the conditional logit model allows these correction components to be interpreted as the likelihood that certain types of households select a *certain type of neighborhood* instead of the likelihood of selecting *a specific neighborhood*.

### Neighborhood Effects Models Incorporating Neighborhood Selection

The neighborhood-effect models estimate the effect of neighborhood income, the share of non-Western minorities, and the share of social housing on individual income from work in 2013. We model the income for all employed persons in 2013 based on neighborhood characteristics in 2010. We compare three neighborhood-effects models: (1) a model without controls, (2) a model controlling for personal characteristics, and (3) a model with correction components for neighborhood types derived from the selection model in Step 1. Both the personal characteristics and the correction components are measured at the individual level; therefore, we use clustered standard errors to account for the nonrandom distribution of individuals across neighborhoods.

## Results

### Descriptive Statistics

Tables 1 and 2 present the descriptive statistics for the neighborhood-level and individual variables included in the selection models, respectively. The average housing value in 2008 was 291,000 euros. The average neighborhood was 3.9 km from a train station, had 76.2 restaurants within walking distance, and was 2 km from a highway. In the average neighborhood, 30 % of homes were social housing, 14 % of homes were built in the past 10 years, 41 % of residents were single, and 12.5 % of residents were non-Western minorities. The majority of individuals in the analytic sample were native Dutch, slightly more than one-half were single, approximately 35 % were younger than age 25, and the average household income was 47,000 euros in 2010.

**Table 2:** Descriptive statistics: Personal characteristics (as of January 1, 2010, selection model), *N* = 24,014

	*N*	%
Ethnicity		
Native Dutch	17,283	72
Non-Western minority	4,258	18
Western minority	2,473	10
Household Type		
Couple with children	4,301	18
Couple	5,572	23
Single or other	14,141	59
Age		
<25	8,574	36
25–65	13,725	57
>65	1,715	7
Gross Household Income (× 1,000) (mean euros)	47.1	
SD	45.8	

Tables 3 and 4 present the descriptive statistics for the variables included in the neighborhood-effect models. These models are estimated on only people who were employed on January 1, 2013. Their average monthly income from work is 3,258 euro. People were, on average, 31 years old; 20 % were living with a partner and children, and 27 % were living with a partner; 9 % were Western minorities, and 14 % were non-Western minorities.

**Table 3:** Descriptive statistics: Individual characteristics (effects model), *N* = 13,430

	Mean	SD	Min.	Max.
Dependent Variable				
Ln (income from work) 2013^a^	7.88	0.650		
Personal Characteristics				
Moroccan	.039		0	1
Turkish	.023		0	1
Surinamese	.022		0	1
Antillean	.010		0	1
Other non-Western	.046		0	1
Western	.093		0	1
Couple	.271		0	1
Couple with children	.200		0	1
Age	30.82	9.00	15	78

^a^We are not able to show minimum and maximum incomes because of Statistics Netherlands disclosure restrictions.

**Table 4:** Descriptive statistics: Neighborhood characteristics (effects model), *N* = 200

	Mean	SD	Min.	Max.
Average Income	23.69	5.52	7.5	46.7
Share of Social Housing	.31	.24	0	1
Share of Non-Western Minorities	.13	.12	0	79

### Modeling Neighborhood Selection

Table 5 presents the results from the conditional logit model based on the full choice set, in which individual and neighborhood characteristics are interacted to predict neighborhood choice. Most of the parameter estimates from the resulting 11 sets of interactions are significant, demonstrating pronounced differences among ethnic groups, household types, age groups, and income groups in the effects of neighborhood characteristics on neighborhood choice. For example, non-Western minorities were the most likely to select neighborhoods with a high percentage of minorities. Similarly, families and those older than 65 were less likely to select a neighborhood with a high percentage of non-Western ethnic minorities than single people and those younger than 65. Based on these individual and neighborhood characteristics, we can only partially explain exactly which neighborhood people select. As argued earlier, many neighborhoods are similar in dwelling values, housing market composition, accessibility, household composition, and ethnic composition. People have a preference with regard to a certain neighborhood type, and whether people select one neighborhood over a similar neighborhood will partly be based on coincidence or on other unmeasured neighborhood characteristics.

**Table 5:** Neighborhood selection model based on interactions between personal characteristics and neighborhood characteristics, *N* = 24,014

	β	*p*
Interactions With Average Dwelling Values		
Non-Western minority	–0.0048	.000
Western minority	–0.0015	.000
Couple	–0.0043	.000
Couple with children	–0.0026	.000
Young (<25 years)	–0.0023	.000
Old (>65 years)	–0.0011	.001
Household income	0.0000	.013
Interactions With Number of Restaurants <3 km		
Non-Western minority	–0.0012	.000
Western minority	0.0002	.508
Couple	0.0000	.903
Couple with children	–0.0030	.000
Young (<25 years)	0.0007	.000
Old (>65 years)	–0.0066	.000
Household income	0.0000	.000
Interactions With Distance to Train Station		
Non-Western minority	–0.0443	.000
Western minority	–0.0482	.000
Couple	–0.0442	.000
Couple with children	–0.0399	.000
Young (<25 years)	–0.0836	.000
Old (>65 years)	–0.0873	.000
Household income	–0.0010	.000
Interactions With Distance to Highway Access Lane		
Non-Western minority	0.0571	.029
Western minority	–0.0345	.260
Couple	0.0564	.013
Couple with children	0.0642	.008
Young (<25 years)	–0.1307	.000
Old (>65 years)	–0.0311	.314
Household income	–0.0010	.000
Interactions With Share of Building Built After 2000		
Non-Western minority	0.0034	.000
Western minority	0.0002	.868
Couple	0.0010	.146
Couple with children	0.0010	0.128
Young (<25 years)	0.0037	0.000
Old (>65 years)	0.0041	0.000
Household income	0.0001	0.000
Interactions With Share of Non-Western Minorities		
Non-Western minority	3.6129	.000
Western minority	1.3285	.000
Couple	0.4052	.014
Couple with children	–0.1690	.365
Young (<25 years)	0.1190	.304
Old (>65 years)	–0.6407	.012
Household income	0.0113	.000
Interactions With Share of Western Minorities		
Non-Western minority	2.8926	.000
Western minority	1.9160	.003
Couple	1.7145	.004
Couple with children	4.8522	.000
Young (<25 years)	–3.8600	.000
Old (>65 years)	–1.4014	.190
Household income	0.0205	.000
Interactions With Share of Social Rented Dwellings		
Non-Western minority	0.0037	.003
Western minority	–0.0067	.000
Couple	0.0007	.554
Couple with children	0.0120	.000
Young (<25 years)	–0.0110	.000
Old (>65 years)	0.0078	.000
Household income	–0.0001	.000
Interactions With Share of Private Rental Dwellings		
Non-Western minority	0.0111	.000
Western minority	–0.0054	.073
Couple	0.0050	.029
Couple with children	0.0143	.000
Young (<25 years)	–0.0140	.000
Old (>65 years)	0.0202	.000
Household income	0.0000	.094
Interactions With Share of Singles		
Non-Western minority	–0.0004	.868
Western minority	0.0131	.000
Couple	–0.0182	.000
Couple with children	–0.0371	.000
Young (<25 years)	0.0326	.000
Old (>65 years)	0.0137	.000
Household income	–0.0001	.002
Interactions With Share of Couples		
Non-Western minority	0.0250	.000
Western minority	–0.0010	.875
Couple	0.0212	.000
Couple with children	0.0115	.023
Young (<25 years)	–0.0504	.000
Old (>65 years)	0.0358	.000
Household income	0.0002	.000
Log-Likelihood		–119,218
Log-Likelihood 0-Model		–127,591
Likelihood Ratio (chi-square test statistic)		16,747
Pseudo-*R*^2^		.0656

### Estimating Neighborhood Effects on Income With Correction for Neighborhood Selection

Table 6 presents the parameter estimates from the regression model predicting log transformed individual earnings as a function of neighborhood income, percentage of social housing, and percentage of non-Western minorities. The first model presents the baseline effect of neighborhood characteristics on individual earnings. This model reveals a small but statistically significant relationship between average neighborhood income and individual income: a €1,000 increase in average neighborhood income is associated with an expected 2 % increase in the annual salary of neighborhood residents. Neither the percentage of social housing nor the share of non-Western minorities emerge as significant predictors of earnings.

**Table 6:** Neighborhood effects on individual income, *N* = 14,340

	Model 1		Model 2		Model 3	
	β	*p*	β	*p*	β	*p*
Neighborhood Characteristic						
Average income (×1,000)	0.022	.000	0.015	.000	0.007	.003
Share social housing	–0.189	.069	–0.179	.005	–0.058	.232
Share non-Western minorities	0.151	.352	0.164	.135	0.021	.765
Personal Characteristics						
Ethnicity (ref. = native)						
Moroccan			–0.201	.000		
Turkish			–0.160	.000		
Surinamese			–0.230	.000		
Antillean			–0.232	.000		
Other non-Western			–0.221	.000		
Western			–0.084	.000		
Household (ref. = single)						
Couple			0.180	.000		
Couple with children			0.083	.000		
Age			0.133	.000		
Age, squared			–0.002	.000		
Instruments						
Component 1					0.037	.000
Component 2					0.052	.000
Component 3					–0.040	.000
Component 4					0.000	.908
Component 5					–0.001	.856
Component 6					0.008	.026
Component 7					0.036	.000
Component 8					–0.025	.000
Intercept	7.423	.000	5.110	.000	7.743	.000
*R* ^2^		.0416		.2028		.3478
*F*		24.10		96.25		438.22

The second model (Table 6, Model 2) introduces the individual-level covariates. The model shows that ethnic minorities have significantly lower incomes than natives. The household composition dummy variables show that household heads in couples and in couples with children have significantly higher incomes than singles. The parameter estimates for the age variables show that income first increases and then decreases with age. Controlling for individual characteristics reduces the parameter estimate for the average neighborhood income on personal income by 31.8 % [(.022 – .015) / .022]; however, it remains statistically significant, suggesting that the association between neighborhood income and individual earnings can be partially explained by the personal characteristics of households most likely to live in areas with a certain income composition. Interestingly, the inclusion of the personal characteristics reveals a suppression effect: the parameter estimate for share of social housing in the neighborhood becomes significant, suggesting that heads of household who moved to a neighborhood with high shares of social housing in 2009 had a lower income in 2013. The suppression effect is likely driven by the inclusion of ethnicity in the model. Native-born Dutch tend to have lower incomes when they live in areas with a high concentration of social housing. Conversely, social housing concentration has a protective function for minorities, perhaps because of the presence of informal networks that aid in securing employment, thus increasing earnings over time. The effect of the percentage of non-Western ethnic minorities remains nonsignificant. The inclusion of the individual-level characteristics provides a significantly better fit to the model than the baseline model with the neighborhood characteristics alone (*R*^2^ = .2028; *F* = 322.4; *p* < .001).

Model 3 substitutes the individual-level characteristics for the eight correction components derived from the neighborhood-selection model. Assuming that selection processes are at play, the parameter estimates for correction components should emerge as statistically significant, and their inclusion in the model should reduce the magnitude of the coefficients for the neighborhood-level variables. Indeed, six of the eight correction components emerge as statistically significant predictors of income, further supporting the contention that people select into neighborhoods at least partially based on shared characteristics that will ultimately bear on their later earnings. In other words, residential preferences are strongly correlated with income.

Perhaps more importantly, the inclusion of the correction components attenuates the effects of both social housing concentration and average neighborhood income on individual earnings. The inclusion of the correction components reduces the effect of average neighborhood income, decreasing the magnitude of the parameter estimate by 68.2 % ((.022 – .007) / .022); however, the coefficient retains statistical significance. This indicates that although much of the relationship between neighborhood income composition and individual earning can be attributed to the differential sorting of households into neighborhoods, neighborhood income still has a residual effect on individual earnings. Put more simply, poor people indeed move to poor neighborhoods, but moving to impoverished neighborhoods further dampens future earnings potential. The inclusion of the correction components provides a better fit to the data than the baseline model (*R*^2^ = .3478; *F* = 788.06; *p* < .001).

## Conclusions

One of the most significant challenges confronting neighborhood effects scholars concerns the assorted issues with neighborhood selection. Households are not randomly distributed across urban areas; rather, they choose a type of neighborhood based on their preferences and their income. Such nonrandom allocation of households across neighborhoods makes it difficult to establish causal relationships between neighborhood characteristics and individual outcomes. Whereas most of the literature sets out to control for selection effects, either through covariate controls or counterfactual models, we argue that the processes through which certain households decide to move to certain types of neighborhoods should be examined and explicitly incorporated in models of neighborhood effects (see also Hedman and Galster 2011; Hedman and van Ham 2012; Ioannides and Zabel 2008; Sari 2012).

In this article, we present an empirical framework to help disentangle selection processes in empirical models of neighborhood effects. We build on prior research by modeling neighborhood choice using a conditional logit model and subsequently incorporating correction components into a neighborhood-effects model of individual income from work. In the first step, we model neighborhood selection for all movers and generate the conditional probability that each head of household would select a certain neighborhood from a choice set of 203 neighborhoods in the Utrecht urban region. In line with previous research, we find that the neighborhood selection process is highly structured and that households are likely to prefer neighborhoods where the population composition matches their own social and demographic background.

In the second step, we model the effect of three neighborhood characteristics on individual income from work, including correction components for neighborhood selection into types of neighborhoods in our model. This approach crucially diverges from prior research in that we construct correction components based on neighborhood types instead of individual neighborhoods, which we argue to be both conceptually and methodologically more appealing. And in our approach, in order to construct correction components based on neighborhood types, we use the full choice set of available neighborhoods in the regional housing market instead of a random choice set. We find that the effect of the average neighborhood income on individual income is reduced when the neighborhood selection mechanism is controlled for. In addition, we find that the model with correction terms explains the variation in the data much better than the standard models.

The conclusion from our models is that controlling for neighborhood selection leads to less biased estimates of neighborhood effects. But most importantly, even after neighborhood selection is controlled for, we still find a small but statistically significant negative relationship between living in a deprived neighborhood and individual income. This is an important finding given that our results suggest that neighborhood effects reflect more than the shared characteristics of neighborhood residents; place of residence partially determines economic well-being.

## Appendix

Our selection model uses the full choice set of alternative neighborhoods and, consequently, the full range of 203 predicted probabilities. Ioannides and Zabel (2008) used a choice set determined by the chosen neighborhood in which the household lived plus a sample of 10 alternative census tracts, randomly selected from all census tracts of the metropolitan area. This resulted in a choice set of 11 tracts (10 of which were random). Prior research has suggested that this approach provides an effective means of estimating neighborhood selection (see Hedman et al. 2011). For the selection model, it does not matter whether a random or the full choice set of neighborhoods is used: the outcomes of the selection model are identical. To support this argument, we estimate two similar neighborhood selection models based on both a full choice (FC) set and on a random choice (RC) set. For comparability, we include all 203 neighborhoods in the RC model; however, the order of the neighborhoods is randomized within individuals, similar to the approach used by Ioannides and Zabel (2008) and Hedman et al. (2011). As might be expected, the outcomes of the two selection models are identical.

However, the choice for an RC or FC set has significant implications for the construction of the neighborhood choice correction terms. When using a RC set in the selection model, the model generates predicted probabilities that a household moves to any of the randomly determined neighborhoods. In the case of Ioannides and Zabel (2008), these were 10 random neighborhoods and the neighborhood the household actually moves to, so a choice set of 11 neighborhoods. When these 11 predicted probabilities are used to construct correction terms, the problem arises that the underlying neighborhoods in each of these correction terms are randomly distributed. So, if the selection model is estimated on an RC set, the first correction term represents for every individual the likelihood of selecting the first random neighborhood. For every individual, this will be a *different* randomly selected neighborhood, with different neighborhood characteristics. Random neighborhood 1 might be attractive to one household because of the relative low dwelling values and to another household because of the relatively high dwelling values. If the selection model is estimated on the FC set, the first correction term represents for every individual the likelihood of selecting the *same* neighborhood. Based on the characteristics of this first neighborhood (e.g., average dwelling values, accessibility, and ethnic composition), the likelihood of selecting this particular neighborhood will be high for certain people and low for others. Therefore, it is possible to control for neighborhood selection by including these correction terms based on the FC set in the neighborhood-effects model. We argue that predicted probabilities based on an RC set are not effective to control for neighborhood selection.

As support for our argument that we need to use the FC set of all neighborhoods in the Utrecht region in our selection model to construct meaningful correction components for our neighborhood-effects model, we compare the effects of correction terms based on the FC set and the RC set. For comparability, we also use the full set of 203 neighborhoods for the RC set, but we randomize them for each household so that the ordering of neighborhoods is different for each household in the model (compared with the FC models, in which the order of neighborhoods is the same for each individual). Then we use PCA to calculate correction components based on the RC selection model. Although PCA on the correction terms from the FC model yields eight principal components, PCA on the correction terms from the RC models yields 99 principal components with eigenvalues greater than 1. Including the eight correction components based on the FC selection model in the model of neighborhood effects significantly improves the model fit, as reported in the section Estimating Neighborhood Effects on Income With Correction for Neighborhood Selection (*R*^2^ = .3478; *F* = 788.06; *p* < .001). However, inclusion of the 99 correction components based on the RC selection model in the model of neighborhood effects does not lead to a significant improvement of the model (*R*^2^ = .0518; *F* = 1.45). Although including the correction components of the selection model based on the FC set leads to much smaller neighborhood effects on individual income, the 99 correction components of the RC selection model barely change the size of the neighborhood effects. We therefore argue that the likelihood of selecting into a random neighborhood is not an effective control for neighborhood selection, and therefore correction components based on the FC set should be used.

## References

[CR1] Bolster A, Burgess S, Johnston R, Jones K, Propper C, Sarker R (2007). Neighbourhoods, households and income dynamics: A semi-parametric investigation of neighbourhood effects. Journal of Economic Geography.

[CR2] Boschman S (2015). Selective mobility, segregation and neighbourhood effects.

[CR3] Boschman S, van Ham M (2015). Neighbourhood selection of non-Western ethnic minorities: Testing the own-group effects hypothesis using a conditional logit model. Environment and Planning A.

[CR4] Bråmå Å (2006). “White flight”? The production and reproduction of concentration areas in Swedish cities 1990–2000. Urban Studies.

[CR5] Cheshire P (2007). *Segregated neighbourhoods and mixed communities: A critical analysis* (Report).

[CR6] Clark WAV, Ledwith V (2007). How much does income matter in neighbourhood choice?. Population Research and Policy Review.

[CR7] Desmond M (2016). Evicted: Poverty and profit in the American city.

[CR8] Durlauf S, Henderson JV, Thisse JF (2004). Neighborhood effects. Handbook of regional and urban economics: Cities and geography.

[CR9] Ellen IG, Turner MA (1997). Does neighbourhood matter? Assessing recent evidence. Housing Policy Debate.

[CR10] Galster G (2002). An economic efficiency analysis of deconcentrating poverty populations. Journal of Housing Economics.

[CR11] Galster GC, van Ham M, Manley D, Bailey N, Simpson L, Maclennan D (2012). The mechanism(s) of neighbourhood effects: Theory, evidence, and policy implications. Neighbourhood effects research: New perspectives.

[CR12] Harding DJ (2003). Counterfactual models of neighborhood effects: The effect of neighborhood poverty on dropping out and teenage pregnancy. American Journal of Sociology.

[CR13] Heckman JJ (1979). Sample selection bias as a specification error. Econometrica.

[CR14] Hedman L, Galster G (2011). Neighbourhood income sorting and the effects of neighbourhood income mix on income: A holistic empirical exploration. Urban Studies.

[CR15] Hedman L, van Ham M, van Ham M, Manley D, Bailey N, Simpson L, Maclennan D (2012). Understanding neighbourhood effects: Selection bias and residential mobility. Neighbourhood effects research: New perspectives.

[CR16] Hedman L, van Ham M, Manley D (2011). Neighbourhood choice and neighbourhood reproduction. Environment and Planning A.

[CR17] Hoffman SD, Duncan GJ (1988). Multinomial and conditional logit discrete-choice models in demography. Demography.

[CR18] Ioannides YM, Zabel JE (2008). Interactions, neighborhood selection and housing demand. Journal of Urban Economics.

[CR19] Logan JR, Alba RD (1993). Locational returns to human capital: Minority access to suburban community resources. Demography.

[CR20] Manski CF (1993). Identification of endogenous social effects: The reflection problem. Review of Economic Studies.

[CR21] McFadden D, Zarembka P (1974). Conditional logit analysis of qualitative choice behaviour. Frontiers in econometrics.

[CR22] Nieuwenhuis J (2016). Publication bias in the neighbourhood effects literature. Geoforum.

[CR23] Nieuwenhuis J, Hooimeijer P (2016). The association between neighbourhoods and educational achievement, a systematic review and meta-analysis. Journal of Housing and the Built Environment.

[CR24] Oreopoulos P (2003). The long-run consequences of living in a poor neighborhood. Quarterly Journal of Economics.

[CR25] Quillian, L., & Bruch, E. (2010, April). *Race and class in neighborhood mobility: A conditional logit model of neighbourhood migration*. Paper presented at the annual meeting of the Population Association of America, Dallas, Texas.

[CR26] Sampson RJ, Morenoff JD, Gannon-Rowley T (2002). Assessing “neighborhood effects”: Social processes and new directions in research. Annual Review of Sociology.

[CR27] Sari F (2012). Analysis of neighbourhood effects and work behaviour: Evidence from Paris. Housing Studies.

[CR28] Sermons MW (2000). Influence of race on household residential utility. Geographical Analysis.

[CR29] Sharkey P, Faber JW (2014). Where, when, why, and for whom do residential contexts matter? Moving away from the dichotomous understanding of neighborhood effects. Annual Review of Sociology.

[CR30] van Ham M, Manley D (2010). The effect of neighbourhood housing tenure mix on labour market outcomes: A longitudinal investigation of neighbourhood effects. Journal of Economic Geography.

[CR31] van Ham M, Manley D (2012). Neighbourhood effects research at a crossroads. Ten challenges for future research. Environment and Planning A.

[CR32] van Ham M, Manley D, Bailey N, Simpson L, Maclennan D, van Ham M, Manley D, Bailey N, Simpson L, Maclennan D (2012). New perspectives. Neighbourhood effects research: New perspectives.

[CR33] van Ham M, Manley D, Bailey N, Simpson L, Maclennan D (2012). Neighbourhood effects research: New perspectives.

[CR34] Vliegen M (2005). *Grootstedelijke agglomeraties en stadsgewesten afgebakend* [Defining urban agglomerations and urban regions].

[CR35] Vogel M, Porter LC, McCuddy T (2017). Hypermobility, destination effects, and delinquency: Specifying the link between residential mobility and offending. Social Forces.

[CR36] White HC (1971). Multipliers, vacancy chains, and filtering in housing. Journal of the American Institute of Planners.

[CR37] Wilson WJ (1987). The truly disadvantaged.

